# The Relationship Between Tryptophan Hydroxylase-2 Gene with Primary Insomnia and Depressive Symptoms in the Han Chinese Population

**DOI:** 10.4274/balkanmedj.2017.1406

**Published:** 2018-11-15

**Authors:** Feng Mei, Yanfeng Wu, Jin Wu

**Affiliations:** 1 Department of Medical Psychology, The Affiliated Brain Hospital of Nanjing Medical University, Nanjing, Jiangsu, China; 2 Department of Neurology, The Second Affiliated Hospital of Nanjing Medical University, Nanjing, Jiangsu, China

**Keywords:** Depressive disorder, Primary insomnia, TPH2 protein

## Abstract

**Background:**

Insomnia often coexists with depression, and there is compelling evidence for a genetic component in the etiologies of both disorders.

**Aims:**

To investigate the relationship between exonic variant (rs4290270) in the tryptophan hydroxylase-2 gene and primary insomnia and symptoms of depression in Han Chinese.

**Study Design:**

Case-control study.

**Methods:**

This study included 152 patients with primary insomnia and 164 age- and gender-matched normal controls. All patients were investigated by polysomnography for 2 consecutive nights. The depressive symptoms were measured by using a 20-item Zung Self-rating Depression Scale. Sleep quality was assessed with the Pittsburgh Sleep Quality index. The genotypes of the TPH-2 gene polymorphism rs4290270 were determined by the polymerase chain reaction-restriction fragment length polymorphism method.

**Results:**

The genotype distributions of the tryptophan hydroxylase-2 gene polymorphism rs4290270 were in Hardy-Weinberg equilibrium in both patients and controls (p>0.05). The allele and genotype distributions of this variant were comparable between patients and controls in all subjects and between genders (all p>0.05). The impact of rs4290270 on self-rating depression scale score changes was statistically significant (p=0.002), with carriers of the A/A genotype having the highest self-rating depression scale score (mean ± standard deviation: 52.73±12.88), followed by the A/T genotype (50.94±11.29, p=0.35) and the T/T genotype (43.48±7.78, p<0.01), and this impact was more obvious in women (p<0.001).

**Conclusion:**

The tryptophan hydroxylase-2 gene polymorphism rs4290270 may not be a susceptibility locus for primary insomnia in Han Chinese, but it may be a marker of depressive symptoms.

Insomnia constitutes a serious worldwide issue, reaching epidemic proportions and leading to an array of comorbidities, including major depressive disorders and impaired cognitive and physical functions (1,2). A comprehensive meta-analysis of 17 studies with 115.988 subjects indicated that the pooled prevalence of insomnia was 15.0% in China (3). Chronic insomnia affects 6% to 10% of the population, while another 25% of people with insomnia report occasional difficulties with sleep (4). Insomnia and depression are two distinct but overlapping disorders, and they often coexist. About 20% of people with insomnia suffer from major depression (5), yet 69% of people with depression have mild or moderate insomnia (6,7). It is widely recognized that insomnia may serve as a useful warning signal, because of the frequent occurrence of insomnia before a bout of depression strikes (8,9,10,11).

There is compelling evidence for a genetic component in the etiologies of insomnia and depression (12,13). A large panel of genes has been identified in association with sleep disorders through many candidate gene approaches and genome-wide association studies, such as genes encoding regulatory factor X3 (14), period 3 (15), clock circadian regulator (CLOCK) (16,17), and adenosine triphosphate-binding cassette, sub-family C member 9 (18). In particular, serotonin (5-hydroxytryptamine or 5-HT), as one of the most important neurotransmitters, is widely recognized as a promising regulator of insomnia and depression (13,19). Tryptophan hydroxylase (TPH) is a rate-limiting enzyme in the biosynthesis of 5-HT neurotransmission, and it plays a key role in the regulation of the availability, turnover, and function of serotonin. TPH has two isoforms, TPH-1 (expressed in peripheral tissues) and *TPH-2* (expressed predominantly in the brain) (20). Genetic variation in the *TPH-2* gene has recently received much attention because of its potential association with the risk of depression and sleep disturbance (13,21,22). For example, in *TPH-2* knockout mice, serotonin was found to be able to suppress photic and nonphotic inputs that manifest at light-dark transitions and shorten the ultraradian duration of wakefulness and non-rapid-eye-movement sleep (21). Moreover, on the population level, an intron variant, rs12229394, in the *TPH-2* gene was found to be associated with depression and fatigue in women (21), and another synonymous variant, rs4290270, in exon 9 displayed a gender-dependent effect on depression susceptibility (23). However, the association of the *TPH-2* gene with depression in patients with primary insomnia has not yet been reported in the medical literature. To fill this knowledge gap, we tested the hypothesis that the *TPH-2* gene exonic variant rs4290270 may be a susceptibility locus for primary insomnia in Han Chinese and further hypothesized that this variant was associated with depression symptoms in patients with primary insomnia.

## MATERIALS AND METHODS

### Study subjects

Initially, a total of 473 patients were admitted for polysomnography between January 2011 and December 2014, 321 of whom were confirmed to have other sleep-related disturbances, such as obstructive sleep apnea syndrome, generalized anxiety disorder, and major depressive disorders and were excluded from this study. Thus, the final sample consisted of 152 eligible patients [mean age ± standard deviation (SD): 45.32±7.24 years] with primary insomnia, including 100 women and 52 men. All 152 eligible patients fulfilled the following criteria provided by the Diagnostic and Statistical Manual of Mental Disorders, 4^th^ ed: difficulty initiating or maintaining sleep or nonrestorative sleep for ≥6 months and clinically significant distress or impairment due to the sleep disorder (19). All patients were investigated by polysomnography for 2 consecutive nights. In addition, 164 age- and gender-matched normal controls (106/58 women/men, mean age±SD: 48.80±6.31 years) were recruited from their regular physical examination in the local community, and they had no history of any psychiatric illnesses or major physical illnesses according to the Diagnostic Interview Schedule. The study protocol was approved by the ethics committees of Affiliated Nanjing Brain Hospital of Nanjing Medical University and The Second Affiliated Hospital of Nanjing Medical University. Written informed consent was obtained from each subject prior to participation.

### Psychological appraisal in patients

For patients with primary insomnia, depressive symptoms were measured by using a 20-item Zung Self-rating Depression Scale (SDS). The index score ranged from 25 to 100, with a high score indicating a high level of depressive symptoms. Sleep quality was assessed with the Pittsburgh Sleep Quality index (PSQI) (24) within a month before this study. The PSQI contains 19 items that can generate the following 7-component scores [ranging from 0 (“better”) to 3 (“worse”)]: subjective sleep quality, sleep latency, sleep duration, habitual sleep efficiency, sleep disturbances, use of sleeping medication, and daytime dysfunction. The 7-component scores are summed into a global PSQI score (range: 0-21), with higher scores indicating worse sleep quality. A global PSQI score of >5 indicates poor sleep quality, and the satisfactory sensitivity and specificity were 90% and 87%, respectively, when compared with objective clinical and laboratory measures (24).

### Polysomnography recordings

Overnight polysomnography examination was recorded for each study patient. Nocturnal polysomnography measurement and analyses of sleep records were performed according to the same procedure as previously described (25). All patients were requested to sleep in the laboratory for three consecutive nights, namely an adaptation night, a sleep deprivation night, and a night of “recovery” sleep. At the start of the second night, the recording process including whole-night polysomnography before and after sleep deprivation, and every examinee was arranged in a room with the temperature between 20 °C and 26 °C. Accommodation conditions were identical during the three nights. Data were excluded in cases of a normal sleeping state affected by accommodation conditions. polysomnography indexes included (1) sleep latency: the time between lights off and the onset of S1; (2) rapid eye movement, sleep latency; the time between sleep onset and the first rapid eye movement sleep; (3) percentage of stage 1 (S1%), stage 2 (S2%), stage 3 (S3%), and stage 4 (S4%): the proportion of stage 1, stage 2, stage 3, and stage 4, respectively, to total sleep time (26).

### DNA extraction and genotyping

Genomic DNA was extracted from 250 mL ethylene diamine tetraacetic acid-anticoagulated venous blood by using the AxyPrep Blood Genomic DNA Miniprep Kit (Axygen, Union City, CA, USA) according to the manufacturer’s recommendations. The genotypes of the *TPH-2* gene polymorphism rs4290270 were determined by the polymerase chain reaction (PCR)-restriction fragment length polymorphism method as previously reported (23). In detail, this variant was amplified using the primers 5¢- AAT TAT GCA CAG CCC ACC ATT T -3¢ (Forward) and 5¢-TTT AGG CCT GCC ATT TGT TAC C-3¢ (Reverse). The PCR amplification was performed in a PTC-200 MJ Research Peltier Thermal Cycler (Bio-Rad Lab., Massachusetts, USA), and it began with initial denaturation at 94 °C for 5 minutes, then 35 cycles of 94 °C for 40 seconds, 57 °C for 40 seconds, and 72 °C for 40 seconds, followed by a final extension at 72 °C for 10 minutes. The restriction enzyme NdeI (NEB, Shanghai, China) was used to digest the PCR products (37 °C for 12 hours) into 328-bp and 153-bp fragments when the rs4290270-A allele was present. The digested fragments were separated on 2% agarose gels and visualized under ultraviolet light.

### Statistical analysis

Continuous variables were presented as mean±SD and categorical variables as percentages. The two-tailed *t*-test and χ^2^ test were performed to compare differences for continuous and categorical variables, respectively. Deviation from Hardy-Weinberg equilibrium and differences in the allele and genotype distributions of the *TPH-2* gene polymorphism rs4290270 were calculated in the SHEs is program (27). A general linear model was used to examine the effect of rs4290270 genotypes on depressive symptoms, the PSQI test, and the polysomnography index, after adjusting for age, gender, and education. All tests were two-tailed, and the significance level was set at p<0.05. The STATA software program (Version 14.0, StataCorp, TX, USA) was used for statistical analyses. Statistical power was estimated using the PS Power and Sample Size Calculations software (Version 3.0).

## RESULTS

### Baseline characteristics

Table 1 shows the baseline characteristics of 152 patients with primary insomnia and 164 normal controls. The distributions of age, gender, and education were comparable between patients and controls (p>0.05 for all). Data on the SDS, PSQI, and polysomnography indexes were only available in patients.

### Genetic distributions of *TPH-2* gene rs4290270

The genotype distributions of the TPH-2 gene polymorphism rs4290270 met the conditions of Hardy-Weinberg equilibrium in both patients with primary insomnia (p=0.58) and normal controls (p=0.07). The allele and genotype distributions of the *TPH-2* gene polymorphism rs4290270 in all subjects and for each gender are presented in Table 2. In all subjects, the frequency of the rs4290270 mutant T allele was 43.4% in patients and 46.6% in controls, with no statistical significance (p=0.42). In addition, the frequencies of the A/A, A/T, and T/T genotypes of this variant were 30.9%, 51.3%, and 17.8% in patients and 25.0%, 56.7%, and 18.3% in controls, and still there was no detectable significance between the two groups (p=0.49). By gender, there was still no observable significance for both alleles and genotypes of rs4290270 in association with primary insomnia (Table 2).

### The *TPH-2* gene polymorphism rs4290270 and changes in psychological characteristics

The changes in psychological characteristics across rs4290270 genotypes were examined using a generalized linear model after adjusting for age, gender, and education in patients with primary insomnia in all subjects and in each gender (Table 3). In all subjects, the impact of the *TPH-2* gene polymorphism rs4290270 on SDS changes was statistically significant (F test p=0.002), with carriers of the A/A genotype having the highest SDS score (mean±SD: 52.73±12.88), followed by the A/T genotype (50.94±11.29, p=0.35) and the T/T genotype (43.48±7.78, p<0.01). The power to detect a significant difference in SDS score between carriers of the A/A and T/T genotypes was estimated to be 95.4%. No significance was attained for changes in the other psychological characteristics (p>0.05). By gender, only the SDS score was changed significantly across rs4290270 genotypes, especially in women (F test p<0.001) (Table 3). Still, there were no significant differences in other psychological characteristics in either men or women (data not shown).

## DISCUSSION

In this study, we aimed to test the association of the *TPH-2* gene polymorphism rs4290270 with primary insomnia and depression symptoms in Han Chinese. Our findings failed to support the hypothesis that rs4290270 is a susceptibility locus for primary insomnia but indicated a significant association between this variant and depressive symptoms, especially the SDS score, in patients with primary insomnia. Epidemiological studies have identified several risk factors for insomnia, such as older age, depression, drinking, and hyperlipidemia (28,29,30). However, these risk factors cannot fully predict the risk for insomnia. Evidence is mounting suggesting that the development of insomnia is partly under genetic control (31,32). A considerable number of genes and variants have been examined as promising candidates for the risk of insomnia (17,33), while no universal consensus has been reached on a certain variant or gene being consistently related to the risk and development of insomnia. So, to unravel the genetic underpinnings of insomnia still requires continuous exploration, perfection, and refinement. The candidate gene approach is a practical tool in genetic association studies. In the present study, we focused on a rate-limiting enzyme in brain serotonergic synthesis, *TPH-2* (34), as a research candidate to examine the susceptibility of an exonic variant, rs4290270, in its coding gene, *TPH-2*, to the risk of primary insomnia. A large number of studies have supported the association of the *TPH-2* gene with central nervous system disorders, such as depression (23), suicidal behavior (20), and attention-deficit/hyperactivity disorders (35). In this study, however, no hint of a significant association was detected between the alleles and genotypes of rs4290270 and primary insomnia. Many factors may have contributed to this nonsignificant finding. One is that rs4290270 is unlikely to be functional because it is synonymous in nature. Another is that the increased risk conferred by an allele or genotype is small, hence it is of interest to incorporate more variants in the *TPH-2* gene and other candidate genes to examine a possible joint influence on predisposition. The third is the gender-dependent contribution of the *TPH-2* gene polymorphism rs4290270 to insomnia and related comorbidities, as a study by Shen et al. (23) reported that the A-A haplotype of rs4290270 and rs7305115 in *TPH-2* gene was remarkably overrepresented in women with major depressive disorders compared with the corresponding female controls. To yield more information, we have split the genetic data by gender, yet the association of rs4290270 with primary insomnia remained nonsignificant. In spite of the nonsignificant association between the *TPH-2* gene polymorphism rs4290270 and primary insomnia, we found that, interestingly, mutation in this variant was associated with a significant decrease in SDS score in patients with primary insomnia, and importantly, this association was independent of age, gender, and education. In support of this finding, a study by Utge et al. (21) reported a significant association of the *TPH-2* gene polymorphism rs12229394 with depression and fatigue in women. Moreover, as identified by other studies, mutation in the *TPH-2* gene rs4290270 was observed to exert a significant impact on *TPH-2* mRNA expression in the postmortem human pons (34,36), which may, at least in part, sustain the involvement of *TPH-2* in neuropsychiatric disorders, including insomnia. In view of these observations, it is reasonable to speculate that the *TPH-2* gene polymorphism rs4290270 may be a marker of depressive symptoms in patients with primary insomnia.

Several limitations of this study should be acknowledged. First, this was a retrospective cross-sectional study that cannot demonstrate causality between the *TPH-2* gene and primary insomnia. Second, the sample size involved in the present study was not sufficiently powerful to detect a modest or small genetic effect. Further confirmation of our findings in an independent and large sample of the Chinese population is warranted to clarify the role of the *TPH-2* gene polymorphism rs4290270 in insomnia. Third, only one variant in the *TPH-2* gene was genotyped, which prevented further locus-to-locus and gene-to-gene analyses, as it is generally accepted that insomnia is a polygenic disorder. Fourth, only Han Chinese subjects were included in this study, and extrapolation of our findings to other nationalities or races was only speculative. Last but not least, although a preliminary association between the *TPH-2* gene polymorphism rs4290270 and depressive symptoms was found in this case-control study, we agree that further confirmation of this association in other cohorts with large samples is necessary. Our findings indicate that the *TPH-2* gene polymorphism rs4290270 may not be a susceptibility locus for primary insomnia in Han Chinese but may be a marker of depressive symptoms, especially SDS score, in patients with primary insomnia. In view of the above limitations, the findings presented here should be considered as preliminary; additional investigations focusing on gene-to-gene or gene-to-environment interactions and seeking to elucidate the molecular mechanisms whereby genetic variation in the *TPH-2* gene and other candidate genes are involved in the pathogenesis of insomnia and depression are needed in the future.

## Figures and Tables

**Table 1:**
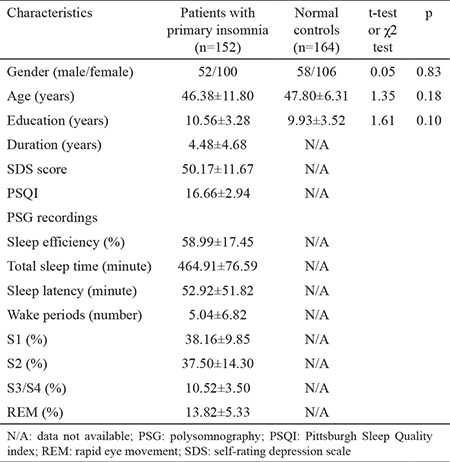
Baseline characteristics in patients with primary insomnia and normal controls

**Table 2:**
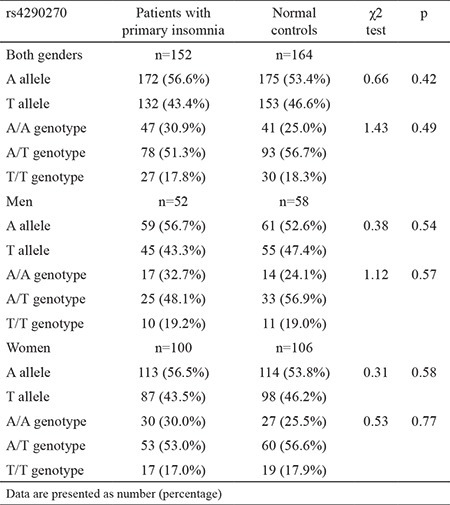
Allele and genotype distributions of the TPH-2 gene polymorphism rs4290270 between patients with primary insomnia and normal controls

**Table 3:**
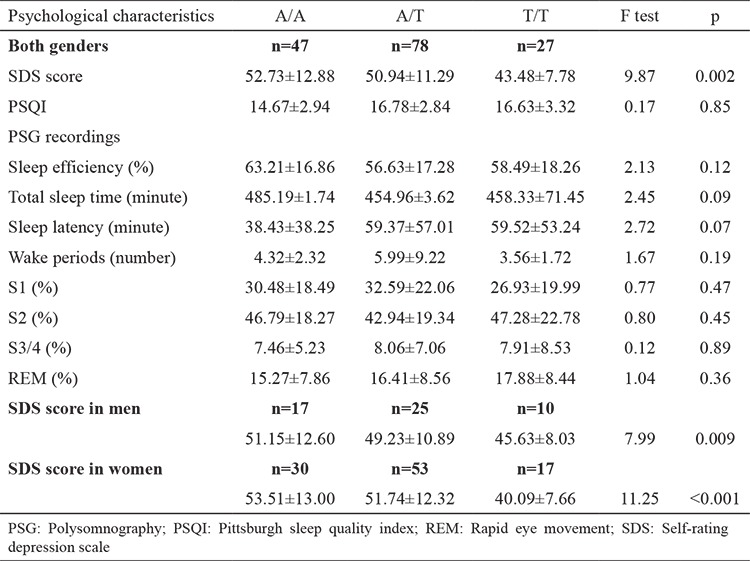
Changes in psychological characteristics across rs4290270 genotypes in patients with primary insomnia
